# Quo Vadis Anesthesiologist? The Value Proposition of Future Anesthesiologists Lies in Preserving or Restoring Presurgical Health after Surgical Insult

**DOI:** 10.3390/jcm11041135

**Published:** 2022-02-21

**Authors:** Krzysztof Laudanski

**Affiliations:** 1 Department of Anesthesiology and Critical Care, University of Pennsylvania, Philadelphia, PA 19104, USA; klaudanski@gmail.com; Tel.: +1-215-662-8000; 2 Leonard Davis Institute for Healthcare Economics, University of Pennsylvania, Philadelphia, PA 19104, USA; 3 Department of Neurology, University of Pennsylvania, Philadelphia, PA 19104, USA

## 1. Introduction

This Special Issue of the *Journal of Clinical Medicine* is devoted to anesthesia and perioperative care. While it is the glazed through aspect of surgical care, it is considered one of the most spectacular achievements of modern medicine. The scope of JCM’s Special Issue intends to highlight the debate regarding the role of anesthesia and the anesthesiologist in perioperative care and how future anesthesiologists can add new value to patient care.

## 2. New Paradigm of Anesthesia Care

**Figure 1:**
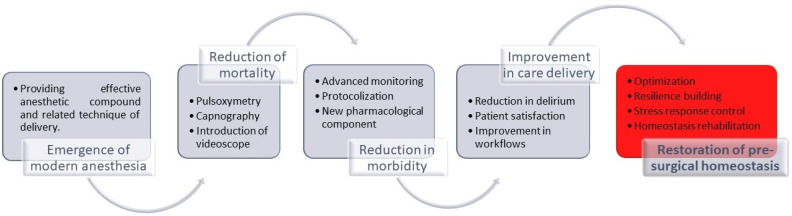
Anesthesia historical milestone.

The ability to take away the surgical patient’s consciousness and pain while rendering him or her motionless in a state of temporary amnesia has enabled great progress in surgery, imaging, and diseases prevention (Figure 1) [1]. Increased safety in delivering anesthesia has also made undeniable progress over the last 40 years. Between 1940 and 2020, anesthesia-related mortality was reduced from 1:1000 to 1:1,000,000. The invention of pulse oximetry, capnography, ultrasound, development of short-acting and liver- or renal-independent medications (esmolol, remifentanil, etc.), and a novel pharmacological method to reverse anesthesia (sugammadex) are advances behind the more modern successes of anesthesia (Figure 1) [2,3,4,5,6]. Improved workflows, safety procedures (checklists, simulations), and maintaining a high level of professional competency have also had a pivotal role in improving outcomes [7,8,9]. The drop in mortality is so profound that studies investigating anesthesia mortality are virtually impossible, with most of the current data published in case series or audits of legal cases [10,11,12]. A decrease in intra-operative morbidity accompanies decreased mortality [12,13,14,15,16,17]. The next milestone for anesthesia in developed countries is debatable, but minimizing maladaptive allostatic sequela after peri-surgical stress seems impactful, achievable, and uniquely positioned to be pursued by next generation of anesthesiologists (Figure 1).

Surgical procedures and related events can result in profound disturbances in homeostasis [18]. The physiological mechanisms designed to prevent or minimize potential damage during normal life may be superfluous during surgery. Their activation results in a less favorable outcome unless suppressed by anesthesia. For example, pain sensation triggers sympathetic discharge and the fight or flight reaction. However, during surgery, sympathetic activity may result in cardiomyopathy and other complications. Anesthesia partially abolishes this effect yet at the cost of side effects [19]. Concomitantly, surgical trauma induces a profound adaptive response, affecting several layers of bodily functioning. Most of these responses are adaptive, but they may lead to undesirable outcomes in certain circumstances. For example, activating the immune system is critical to healing. It also causes collateral damage, resulting in organ failure and increased hypercoagulability [20,21]. Finally, the anesthetic used may have an adverse effect during peri-operative period, resulting in neurotoxicity and potential neurodegeneration [22]. 

It is becoming increasingly apparent that regaining preoperative homeostasis during the postsurgical period is impossible in some individuals [19,20]. The postsurgical processes are different from the primary pathological process that leads to surgery as they center around surgical insult and anesthesia. For example, immune system activation resembles sterile inflammation, not a septic process [23,24,25,26,27,28]. Surgery involves significant tissue destruction, blood loss, alteration in microbiota with the possible leak of the inflammatory pathogen-associated molecular pattern (PAMP), and the induction of catabolism [26,29,30,31,32,33,34,35,36]. Similar abnormalities are seen in other critical care illnesses, including traumatic brain injury, COVID-19, ARDS, stroke, and acute coronary syndrome. However, some important differences exist. The postsurgical state may extend beyond immediate recovery and the postoperative period, lasting months or representing a new allesotatic state [28,37,38,39,40]. Consequently, an individual cannot regain preoperative health or homeostasis. Alternatively, the newly acquired process remains in place long enough to contribute to increased morbidity after several years, even if the change eventually resolves [41]. Allostasis assumes the emergence of a new adaptive balance, which may be beneficial or detrimental to the long-term health of the individual (Figure 1) [37,38]. Epigenetic mechanisms, miRNA, acquired autoreactivity, metabolic shifts, and the persistent subcortical changes in the central nervous system responsible for homeostasis are underpinning mechanisms that lead to persistent post-surgical sequelae and allostasis [21,36,42,43,44,45,46,47,48,49,50]. Individuals with pre-existing comorbidities and disadvantageous socia-economical backgrounds will be more vulnerable to the emergence and persistence of unfavorable allostasis [51]. 

**Figure 2:**
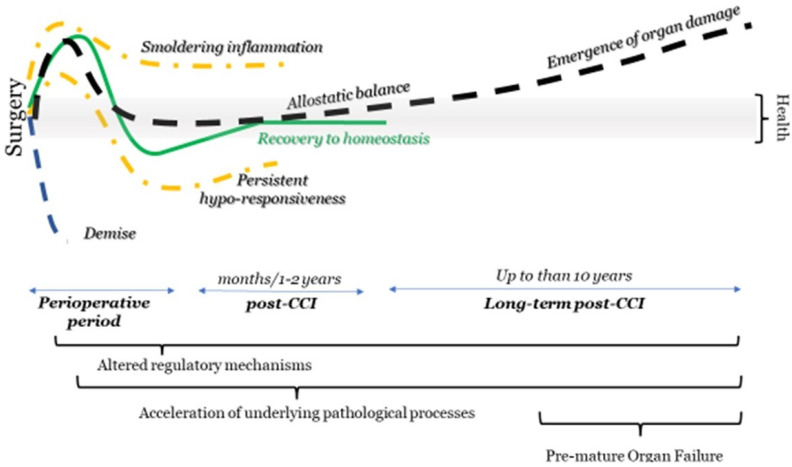
Presurgical stress can trigger different trajectories among patients, including maladaptive allostasis balance leading to long-term consequences.

The recovery trajectory depends on the inherited features of the patient and the nature and magnitude of the stressor (Figure 2). Even a single incident of anesthesia, surgery, or other critical grade insults may reverberate for months and even years [27,32,33,39,42]. These parameters are modifiable during the post- and perioperative period, placing anesthesiologists in a unique position to foster restoration of presurgical health.

## 3. The Effect of Anesthesia on Long-Term Postoperative Outcomes

The idea that anesthetic management affects long-term outcomes is often debated but relatively few new studies of high quality have been published. The short duration of anesthesia and the predominance of surgery-driven insult link the latter to long-term unfavorable declines [52]. Initial enthusiasm was quickly dampened by conflicting data, showing the variable effects of various anesthesia techniques on long-term outcomes with the almost “classical” failure of experimental data to yield a consistent clinical practice adoption [53,54,55,56,57]. Few studies are reviewed below to demonstrate methodological difficulty in proving the point.

The application of regional anesthesia seems to be advantageous as compared to general anesthesia via several mechanisms. First, it reduces inflammation and sympathetic discharge while providing more effective oxygenation that in turn implies a lack of hypotension [58]. However, translating the hypothetical and bench-driven benefits to the clinical realm is not straightforward despite several areas of potential benefits [59]. Poor quality studies and insufficient understanding of the interaction between cancer and anesthesia may be at fault [60,61]. The highly debated question of regional anesthesia reducing metastasis recurrence long-term is difficult to analyze given the plethora of studies that fail to provide clear benefits of using regional anesthesia despite the importance of the question [52,53,54,55,56,57,58,59,60,61,62,63,64]. Remarkably, the depth of anesthesia correlates in some studies with the emergence of postoperative delirium [65,66,67]. The emergence of delirium carries a significant risk of long-term decline, but definitive studies are missing. One study suggests that a singular dose of clonidine changes mortality for several years to come, but metanalysis does not confirm these initial observations [68,69,70]. Nevertheless, the introduction of dexmedetomidine reinvigorates the field with several groups, suggesting long-term protective benefits [71,72]. Interestingly, some authors suggest that dampening the initial sympathetic response results in a more effective resolution of the inflammation with potential long-term benefits. Still, large studies failed to demonstrate the benefit of pharmacological sympathectomy on outcomes delayed by years. There is a need to appreciate the extraordinary methodological obstacles to be overcome while studying the long-term effect of anesthesia.

Despite the lack of clear advantages of different strategies to execute anesthesia plan on long-term outcomes, interest should not abate. The application of the anesthesia technique has to be tailored to the patient’s condition in more detail and toward a resolution, allowing for the personalization of therapy. For example, identifying a particularly vulnerable population may yield tangible long-term benefits [73,74]. What makes this task particularly difficult is complexity of interventions. For example, ketamine has several properties, including a variable effect on the cardiovascular system, sympathetic system activation, and immunomodulation. This nexus of interactions and potential effects results in the difficult application of an anesthesia compound to precisely defined clinical targets. Anesthetic agents also have adverse effects on top of the primary desired rendering patients unconscious [75,76]. However, the first step in understanding the delivery of clinical interventions is to provide a standardized was to document interventions. 

## 4. Long-Term Effects Intervention

**Figure 3:**
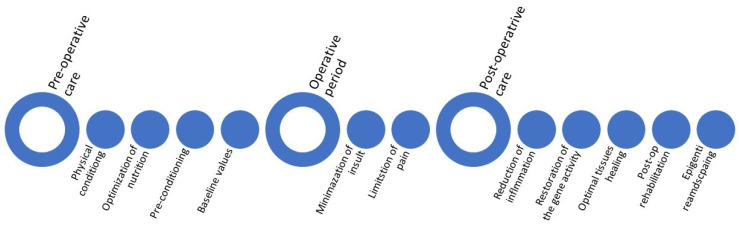
Strategies to improve peri-operative and long-term outcomes.

The lack of clear success in enhancing long-term outcomes in patients undergoing surgery and anesthesia should not sway anesthesiologists from pursuing the goal of restoring patient health to presurgical homeostasis. The anesthesiologist could provide enhanced care before, during, and after surgical insult to restore pre-anesthesia homeostasis in routine cases (Figure 3). 

### 4.1. Preoperative Period

Currently, anesthesiologists focus on perioperative care, often limited to preoperative visits or assessments, the delivery of anesthesia, and immediate recovery. Sometimes, a preoperative clinic allows for more pre-emptive engagement with patients. Such a short engagement makes long-term continuity of care impossible despite providing potential opportunities to prepare the patient for surgery.

The critical task at hand is the optimization of the patient before surgery. This may require a postponement of the surgery, but there is a relative paucity of data to guide optimal timing [77,78]. Some advanced computational techniques (artificial intelligence) may help determine the optimal procedure timing, allowing more time for patient optimization [74]. The same AI algorithm could identify which interventions need to be deployed before surgery, providing the most advantageous edge for the patient in preparation for surgical stress [79].

Methods exist to prepare an individual for surgical stress more resiliently. First in order of importance should be the elimination of any additional and modifiable stressors. Drug abuse, obesity, obstructive sleep apnea, uncontrolled diabetes, or hypertension are potential addressable factors. Encouraging patients to address them will positively impact their outcomes. Adequate nutrition is critical for the individual’s recovery as post-surgical catabolism is inevitable [80,81]. Finally, physical activity is critical before surgery as aerobic exercise positively affects the heart and lungs, prevents weight gain, and is linked to the favorable outcome of anesthesia and surgery. Cognitive engagement also has a positive effect [80,82,83,84]. Engagement of the patients in the process is critical [80]. The barrier may be reimbursement structure.

The nature of the approach increases the individual’s resilience to surgical stress before surgery. Preconditioning is the phenomenon that triggers an adaptive response to significant stress by providing a low-grade trigger before an anticipated major stressor occurs [85,86,87,88]. As a result, this adaptive response is more rapidly deployed in case of stress. Alternative mechanisms dampen the immunological response [89]. A human trial seemed to demonstrate the benefits of this approach when volatile anesthetics were used, but only short-term effects were studied [90]. Many preconditioning techniques involve significant surgical manipulation, but less invasive methods were also suggested, including electroacupuncture or the injection of danger-associated molecular patterns (DAMP) protein [91,92].

Another approach is to proactively mediating the immune system response to mitigate immune system activation in peri-operative time. However, the failure of several cytokine-targeted therapies in several critical care conditions suggests that this approach must be quite selective. One alternative may be to reprogram the innate immune mechanism before the surgery to mitigate potential overactivation without globally suppressing it [93,94,95]. Manipulation with TLR pathways may be particularly beneficial in individuals undergoing procedures with a high risk of pathogen-associated molecular patterns (PAMP) release. Protectin is particularly effective in modulating granulocyte activation [96,97,98]. Finally, inducing M1 to M2 switch or modulating T cell population is another potential pre-emptive strategy [99,100]. Alternative means may involve manipulation with glucose metabolism and inflammation by increasing their resilience to surgical stress [101,102,103].

### 4.2. Operative Period

So far, the primary effect on long-term peri-operative mortality demonstrated that a minimization of the surgical insult by introducing laparoscopic or robotic surgery and shortening the duration of cases in general. The anesthesia management of interoperative care should entail carefully titratable anesthesia at a level tailored to the procedure, utilizing an objective measurement while preventing repetitive hypotension and hypoxemia [104,105,106,107]. However, a more precise definition of hypotension is needed. That definition should be contextualized and personalized to each patient based on observation during the preoperative period [104]. The data to decide which anesthetic is preferable are conflicting. Some studies have demonstrated the benefit of volatile anesthetic, while others are more supportive of intravenous anesthesia [108,109]. Tailoring anesthesia drugs to specific surgery needs is a potential strategy, yet adequate studies need to be conducted. Data on regional anesthesia are also inconclusive but suggest that utilizing regional techniques in elderly patients in pre-existing decline are potentially more beneficial long-term [59,60,63,64]. 

### 4.3. Postoperative Period

Current evidence strongly support that enhanced recovery after surgery (ERAS) philosophy accelerates patients return nominal activity. Inherently, several protocols in ERAS seem to minimize iatrogenic injury despite challenging pre-existing dogmas. Physiologically, ERAS protocol minimizes patient exposure to a challenging hospital environment, focusing on rapid discharge from the hospital. Post-discharge engagement in a rehabilitation plan is critical to minimize long-term decline [110]. Postoperative care has also included the reconciliation of medication to address pre-existing conditions. Two studies demonstrated the interesting effect of diminished frequency of statin intake, suggesting that perception of potential complications may lead to the withdrawal of beneficial medications.

Several studies suggested that surgery results in smoldering inflammation and altered metabolism. The removal of DAMP would be the logical strategy and is close to being deployed clinically [24,26,96]. However, the therapeutic means to address it are somewhat limited as the current diagnostic means to characterize immune system activation are still very constrained. There is a need for more advanced, multidimensional techniques to characterize the post-surgical landscape as it was already demonstrated in trauma [111]. In terms of intervention, several means are available for clinical testing based on already conducted bench research. Lipoxin, annexin, HIF-1, H_2_S, and resolvins are examples of endogenous mediators that could be employed to terminate smoldering inflammation [48,100,112,113,114]. Vagal nerve stimulation seems to be even more reachable, considering their significant utilization in other fields of medicine [115,116]. Finally, overactivated genes can be targeted with genome therapy [117,118]. All these measures should aim at the restoration of presurgical leukocyte status and metabolome. However, a precise understanding of the immune and metabolic systems must first be attained as the immune and metabolic systems are multidimensional and differentially activated [48,49,119].

## 5. Innovation in Service

The implementation of any strategy requires a qualitative change in healthcare delivery. Obamacare, corporatization, and COVID-19 are rapidly accelerating innovation in medicine [120,121]. This is remarkable achievement considering that medicine is a conservative field. Several innovative techniques will challenge current anesthesiology practice, while their adaptation should provide a much-needed qualitative jump to improve long-term outcomes.

AI (artificial intelligence)/CDSS (computer-decision support systems) are increasing in importance in healthcare delivery, resulting in greater investment [122,123,124]. Their current role is mostly focused on radiology and image analysis, but it is only a question of time before they enter the anesthesia world in the domains of monitoring, adverse event prediction, and drug delivery. They can augment the delivery of anesthesia by serving as virtual assistants [125]. Plus, they are pivotal in analyzing long-term continuous data as well as taking into account several variables at the same time. Consequently, a correlation between a truly significant clinical factor and long-term outcome can be uncovered [126]. Hopefully, they will be used to assess the risk of optimal timing for surgery [77]. They can predict what is a critical, pre-emptive intervention to avoid hypotension and hypoxia during surgery [105]. Finally, as a neuronal network, they can be utilized to simulate the effect of suggested therapies on multi-omics physiology, providing a more precise selection of intervention [126,127]. Understanding AI/CDSS intricacies and accepting their guidance will meet resistance. Yet, it is hard to imagine that AI/CDSS will stay away from medicine for long despite initial implementation setbacks [128]. 

Nanotechnology offers a unique forey into the new world of possibilities [129,130]. The most relevant and daring is the example of nanotechnology providing a new way of delivering and maintaining anesthesia to surgical patients [129]. In this Special Issue of JCM, an article is devoted to this prospect. Nanotechnology will be critical in creating a modern way to improve health, deliver medications, and create brain–mind interfaces.

Genomic technologies allow for precise gene manipulation [131]. This may be critical in subduing inflammation post-surgery by either inducing a protective mechanism or supporting a proinflammatory one [132]. The fidelity of CRISP delivers high-resolution gene editing, yet clinical consequences are difficult to predict with AI. mRNA-based gene delivery may be a way to affect the expression of proteins in a safer way. 

Robotics has enjoyed an increased footprint in hospital and surgical theaters. Initially, they were used to augment pharmacy and laboratory services. With the introduction of the DaVinci system, a new era in healthcare was delivered, demonstrating that surgery may be less burdensome and taxing, thus resulting in more favorable outcomes [133,134,135,136]. Surgical robotic systems are revolutionary in surgery, nonetheless, and their introduction in anesthesia is relatively slow [137]. Pharmacological robots utilize a close loop, semiautonomous system to maintain certain parameters by manipulating the delivery of anesthesia under the supervision of an untrained professional. The first commercial system failed secondary to a built-in assumption, as well as engineering and regulatory constraints. However, with the progression of AI, it is only a matter of time before a more sophisticated system will emerge [138]. The pharmacological system provides a close loop medication administration to achieve a certain goal such as arterial pressure or train-of-four with obvious implications for providing hemodynamic stability during surgery. Finally, robots entered the world of physical manipulation by assisting with intubation, regional anesthesia, and intravenous catheter placement [139,140,141].

The brain–computer interface (BCI) is a system that allows for the interaction of electronic devices with the brain. Bi-Spectral monitors can be classified as BCI devices in terms of being non-invasive, capturing filed potential, and producing visual input [142]. Some of these interfaces are “simple devices” implanted in specific areas of the brain aimed at the restoration/augmentation of a function [143]. These devices are of relatively large scale, while direct neuronal-silicone nanoscale junctions are being developed to allow for long-term implantation without compromising the device by the host’s immune mechanisms [144]. Several applications are possible, including the delivery of drugs to discrete areas of the brain, as compensation for function loss, or for better control of prosthetics devices [144,145]. Furthermore, BCI may allow for new ways of inducing anesthesia or the collaboration between providers during anesthesia services by means of direct information exchange in the brain and the utilization of external brainlets. 

Finally, a wide array of strategies to augment human brain functions is suggested to support future anesthesiologist. In fact, many of these innovations were attempted, including the implementation of supercomputing power, direct application of artificial intelligence, and cloud computing, resulting in direct cognitive enhancement of the brain. Most of these innovations are years away from fruition; however, they may dramatically shape the world of anesthesia in the next 20 to 30 years. One of the most vexing ideas is “transparent shadowing”, allowing humans to experience the fully immersive life of other humans with the nano neuronal-robotic-assisted interface. It allows incredible insight into patient experiences for more precise diagnoses [146].

## 6. The Market Value of the New Paradigm of an Anesthesiologist

It is incredibly difficult to predict the job market’s effect on the future skills required by anesthesiologists to stay competitive. However, the US healthcare market is clearly evolving toward providing more value while relentless innovation continues to offer better care to patients. 

Anesthesiologists are highly trained professionals; their job is inexplicably paired with hospital work and the surgical theater, placing them in the center of patient care. However, they come under increased pressure from alternative anesthesia providers, the consolidation of the markets, and the corporatization of medicine in the US [147,148,149]. Propagating anesthesia-related skills outside the operating room or delivering anesthesia by non-residency trained physicians (interventional radiologist, gastroenterologist, certified registered nurse anesthetist, anesthesia assistant, Sedasys^®^) is commonplace and increasing in frequency [138]. Some of these trends are also present outside the US market, such that there is an increasing concern about the future of anesthesiologist-provided care in terms of value as compared to other providers or the future delivery of anesthesia. The current demand for anesthesia services outstrips the supply, yet increased competition may undermine the current paradigm on which current anesthesiologist’s value relies [148,150]. Consequently, future anesthesiologists should look into the specific value they can deliver in perioperative care.

Providing complex, expanded perioperative care aimed at the restoration of presurgical care utilizing several innovative technique should be the ultimate goal of future anesthesiologist. This unique value proposition considers in-depth education, experience, and the available skills for the physician anesthesiologist. This is consistent with the position on the future of anesthesiology expressed by professional leadership despite the meager participation of anesthesiologists in national and regional associations. Focusing on long-term health restoration puts anesthesiologists in a different niche as certified nurse anesthetists or other anesthesia providers.
